# 67 million natural product-like compound database generated via molecular language processing

**DOI:** 10.1038/s41597-023-02207-x

**Published:** 2023-05-19

**Authors:** Dillon W. P. Tay, Naythan Z. X. Yeo, Krishnan Adaikkappan, Yee Hwee Lim, Shi Jun Ang

**Affiliations:** 1 Institute of Sustainability for Chemicals, Energy and Environment (ISCE2), Agency for Science, Technology and Research (A*STAR), 8 Biomedical Grove, #07-01 Neuros Building, Singapore, 138665 Republic of Singapore; 2 Hwa Chong Institution, 661 Bukit Timah Road, Singapore, 269734 Republic of Singapore; 3 National Junior College, 37 Hillcrest Road, Singapore, 288913 Republic of Singapore; 4 Synthetic Biology Translational Research Program, Yong Loo Lin School of Medicine, National University of Singapore, 10 Medical Drive, Singapore, 117597 Republic of Singapore; 5 Institute of High Performance Computing (IHPC), Agency for Science, Technology and Research (A*STAR), 1 Fusionopolis Way, #16-16 Connexis, Singapore, 138632 Republic of Singapore

**Keywords:** Cheminformatics, Combinatorial libraries, Sustainability

## Abstract

Natural products are a rich resource of bioactive compounds for valuable applications across multiple fields such as food, agriculture, and medicine. For natural product discovery, high throughput *in silico* screening offers a cost-effective alternative to traditional resource-heavy assay-guided exploration of structurally novel chemical space. In this data descriptor, we report a characterized database of 67,064,204 natural product-like molecules generated using a recurrent neural network trained on known natural products, demonstrating a significant 165-fold expansion in library size over the approximately 400,000 known natural products. This study highlights the potential of using deep generative models to explore novel natural product chemical space for high throughput *in silico* discovery.

## Background & Summary

Nature produces natural products of immense chemical diversity^1,2^. A vast assortment of molecular scaffolds are produced by organisms to interact with their environment and to engage in chemical warfare with each other. This natural diversity has also been leveraged for wide-ranging applications such as in agricultural pesticides to increase food production^3^, food preservatives to facilitate distribution and storage^4,5^, and most prominently as therapeutic agents to treat diseases^6–8^. Indeed, it has been estimated that approximately 80% of all clinically used antibiotics can trace their origins to a natural product^6^.

**Fig. 1:**
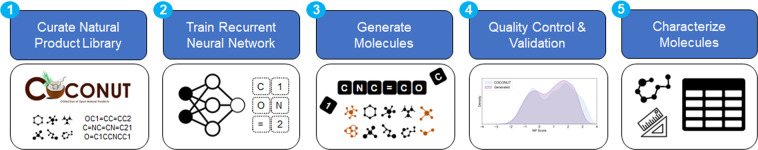
Workflow to generate and characterize a natural product-like compound database using a recurrent neural network trained on known natural products.

Despite nature’s potential for providing valuable molecules, assay-guided natural product discovery has been a low-yielding investment since the golden age of discovery in the 1960s^9^. After the initial wave of uncovering structurally unique and accessible natural product chemical space, subsequent efforts to venture into less accessible chemical space or to “rediscover” known natural product classes for novel applications have been met with limited success^10^. Tremendous effort must be invested in the biosynthesis, curation and characterization of natural product libraries, resulting in the culmination of only ∼400,000 fully characterized natural products known to-date^11^. The significant financial and resource requirements of assay-guided investigations have also resulted in a broad dampening of commercial interest surrounding natural product discovery^12^. However, the advent of deep generative modelling^13^ and high throughput *in silico* screening^14^ presents an opportunity to circumvent traditional time-consuming, costly, and experimentally-driven natural product discovery to reformulate it as a computationally-driven inverse design problem^15^. The potential of such an approach would also scale with the increasing size and availability of natural product databases^16^, growing alongside the trend of digitalization in chemical research^17^. In this data descriptor, we report an expansive, curated database^18^ of 67,064,204 natural product-like molecules generated via an *in silico* pipeline (Fig. 1), representing a significant 165-fold expansion over the ∼400,000 known natural products^11^. We envision *in silico* structural generation playing an integral role in the future of natural product discovery^19^.

In contrast to manually curated natural product libraries, deep generative models transcend the boundaries of human-dependent molecular design to significantly expand chemical search space by orders of magnitude while concurrently reducing financial and resource requirements^20,21^. Some examples of deep generative architectures that have been employed for de novo molecular design include variational autoencoders (VAE)^22,23^, recurrent neural networks (RNN)^24–26^, and generative adversarial networks (GAN)^27–29^, with each adopting a different strategy with their own strengths and weaknesses^30^. The SMILES-based (Simplified Molecular Input Line Entry System)^31^ RNN architecture with long short-term memory (LSTM) units was favoured in this work for its demonstrated ability to robustly generate novel and chemically diverse molecular entities in a low data regime^32^. A systematic benchmarking study^33^ reported that SMILES-based LSTM generated 95.9% valid molecular structures, a significant improvement over VAE (87.0%) and GAN (37.9%) based architectures.

Here, we trained an LSTM model^24^ on tokenized SMILES (with stereochemistry removed) from 325,535 (80%) out of the 406,919 known natural products in COCONUT, the collection of open natural products (https://coconut.naturalproducts.net/, accessed 1 Aug 2022)^11^. The model was able to break down SMILES into unique tokens (e.g. C, N, S, O, c, n, 1, 2..etc), learn how to assemble these token together according to the molecular language of natural products, and generate 100 million natural product-like SMILES with no specified stereochemistry^34^. Although stereochemistry in natural products can confer specific bioactivity^35^, our pipeline removes stereochemistry to reduce data complexity, lower file size, and improve fidelity of the generated structural database. In any case, a range of feasible stereoisomers for each molecule can still be obtained through iterative enumeration of its 3D structures^36,37^ followed by back transformation to stereospecific SMILES^38^. Following this approach, extended isomer libraries of shortlisted SMILES of interest can be generated to cover wider isomeric space than a database of pre-defined stereospecific SMILES.

**Fig. 2:**
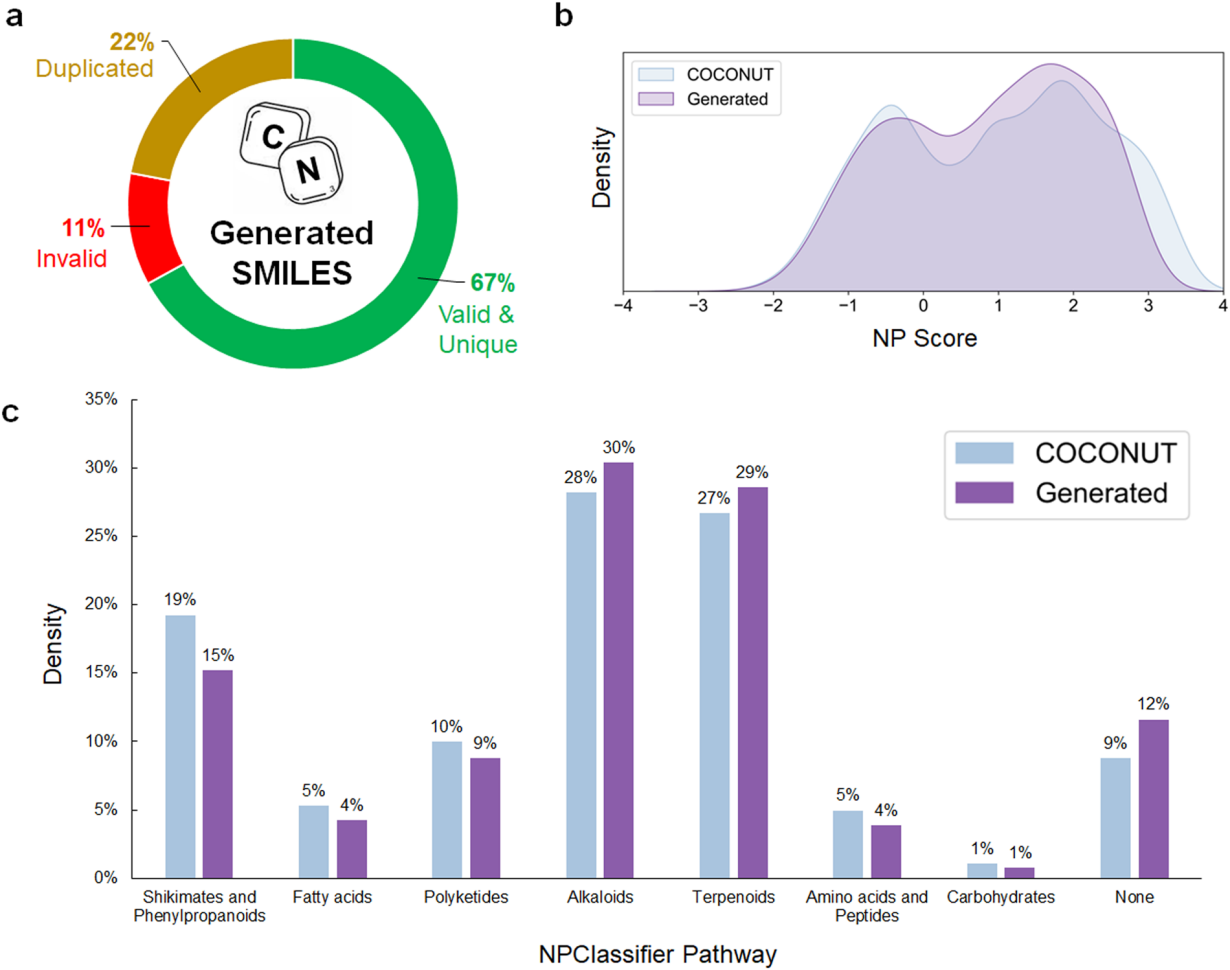
Comparison overview of generated and COCONUT^11^ natural product databases. (**a**) Overview of 100 million generated natural product-like Simplified Molecular Input Line Entry System (SMILES)^31^ generated with trained long short-term memory (LSTM) model. (**b**) Natural product-likeness score (NP Score)^42^ distributions and (**c**) NPClassifier^43^ pathway classifications of valid, unique natural product-like SMILES generated by LSTM model versus known natural product SMILES from COCONUT database^11^. *NOTE: summed percentages may exceed 100% as some molecules have more than 1 label*.

Although alternative approaches for the generation of natural product virtual libraries have been attempted^39,40^, prior libraries have been limited in terms of novelty (frequent re-occurrence of well-known scaffolds)^38^, natural product-likeness (43% meeting threshold compared to 85% in the training set)^39^, and scale (<1.5 million molecules)^39,40^. Moreover, these previously generated natural product virtual libraries have not been publicly released. In this data descriptor, we present an openly available virtual library^18^ of >67 million natural product-like SMILES with a distribution of natural product-likeness scores similar to that of known natural products (Fig. 2) yet encompassing expanded physiochemical and structural space, indicating its potential for *in silico* discovery of natural products.

Cheminformatics toolkits RDKit^36^, ChEMBL chemical curation pipeline^41^, NP Score^42^, and NPClassifier^43^ were employed to sanitize, analyze, and characterize the generated 100 million natural product-like SMILES database (Fig. 2).

First, RDKit^36^ function Chem.MolFromSmiles() was used to filter out 9,596,585 syntactically invalid SMILES from the 100 million generated set. Second, to ensure molecular uniqueness within the dataset, RDKit functions Chem.MolToSmiles(Chem.MolFromSmiles()) and Chem.inchi.MolToInchi() was used to convert the generated SMILES into canonical SMILES and International Chemical Identifier (InChI) representations for comparison and filtering, resulting in the removal of 22,484,883 (22%) duplicates (Fig. 2a). Third, the ChEMBL chemical curation pipeline^41^ was applied for further sanitization and standardization by:

1. Checking and validating chemical structures, assigning an error score if structural issues are detected. Error scores increase with the severity of the problem.
2. Standardizing chemical structures based on FDA/IUPAC guidelines^44^
3. Generating parent structures by removing isotopes, solvents, and salts

Through this process, a further 854,328 invalid molecules with penalty scores exceeding 5 (indicating severe structural issues), were filtered out. Combined with the earlier detected syntactically invalid SMILES, a total of 10,450,913 (11%) invalid generated SMILES were identified and removed (Fig. 2a). The top 2 structural errors reported amongst the remaining valid molecules were (1) undefined stereochemistry (95%), which was due to the generation of SMILES without stereochemistry, and (2) the need for (de)protonation (2%), which was addressed later in Step 3 of the ChEMBL chemical curation pipeline. On the whole, these pre-processing steps refined the initial dataset down to this work’s reported 67,064,204 (67%, Fig. 2a) valid, unique, natural product-like SMILES generated database^18^.

Fourth, RDKit was used to calculate natural product-likeness scores (NP Score)^42^ for both known natural product SMILES and generated SMILES (Fig. 2b). NP Score employs atom-centred fragments (HOSE codes)^45^ and bonding information to characterize structural features and calculate a Bayesian measure of molecular similarity to known natural product structural space^42^. The NP Score distribution of the generated natural product-like SMILES was found to closely resemble that of known natural products from the COCONUT database (Fig. 2b) with a Kullback-Leibler (KL) divergence of 0.064 nats, supporting that natural product-like molecules had been generated.

Fifth, the NPClassifier^43^ toolkit was used to classify both natural product-like SMILES generated from the trained model and known natural product SMILES from the COCONUT database (Fig. 2c). NPClassifier^43^ is a deep learning tool that considers structural features (counted Morgan fingerprints)^46^, taxonomy of the producing organism, nature of the biosynthetic pathway, and biological activity to characterize molecules in a holistic natural product classification framework. Despite this, 7,779,787 (12%) of the generated valid SMILES received no pathway classification – a larger fraction than 35,708 (9%) of the known natural product SMILES that also received no pathway classification. It has been reported^43^ that deficiencies in NPClassifier can be traced back to limitations in its training data as the model relies on existing knowledge of natural products to classify molecules based on structural similarities. The comparatively higher percentage of generated SMILES with no NPClassifier pathway class suggests the presence of either synthetic structural features, or novel natural product class(es). However, similarities in the natural product-likeness score distributions of the generated and known datasets (KL divergence of 0.064 nats) suggests promising potential toward the latter. The remaining 59,284,417 (88%) of the generated valid natural product-like SMILES were annotated with a comparable distribution of biosynthetic pathways as known natural products from the COCONUT database with a KL divergence of 0.047 nats.

Finally, to describe physiochemical space covered by known natural products in the COCONUT database versus the >67 million natural product-like generated database, 10 physiochemical molecular descriptors for each molecule were calculated using RDkit^36^:

1. Number of aromatic rings
2. Number of aliphatic rings
3. Wildman-Crippen LogP (partition coefficient)^47^
4. Molecular weight
5. Number of hydrogen bond acceptors
6. Number of hydrogen bond donors
7. Number of heteroatoms
8. Topological polar surface area (TPSA)
9. Number of rotatable bonds
10. Number of valence electrons

**Fig. 3:**
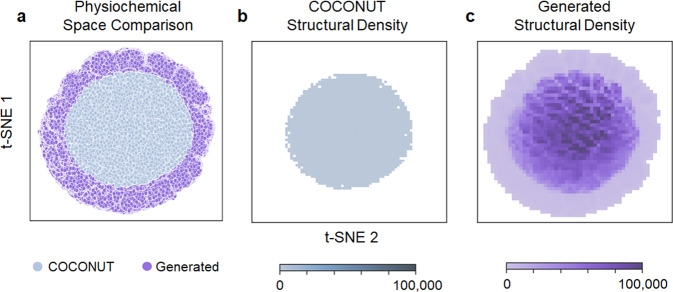
Visualization of expanded physiochemical and structural space afforded by the generated database. (**a**) T-distributed stochastic neighbour embedding (t-SNE) 2D projection of 10 RDkit physiochemical descriptors for 67,064,204 natural product-like structures generated from our trained model and 406,919 known natural product structures from COCONUT, the collection of open natural products^11^. (**b**) Density plot of known natural product structures in t-SNE 2D projected space. (**c**) Density plot of generated natural product-like structures in t-SNE 2D projected space.

T-distributed stochastic neighbour embedding (t-SNE) dimensionality reduction of the 10 calculated molecular descriptors into two-dimensional space was performed and plotted to visualize both physiochemical and structural space coverage (Fig. 3a).

The t-SNE 2D comparison shows a significant increase in physiochemical space covered by generated SMILES (Fig. 3a), indicating the presence of structurally novel natural product-like molecules in the generated database. Density plots (Fig. 3b,c) showing the concentration of structures across the t-SNE 2D projected space also highlight the significantly expanded structural space offered by the generated database even in overlapping physiochemical space (Fig. 3c). Overall, this workflow has enabled us to generate a significantly expanded database^18^ of 67,064,204 characterized natural product-like molecules, greatly increasing natural product chemical space by 165-fold over the currently estimated 400,000 natural products known^11^. The >67 million natural product-like compound database^18^ along with supporting files for the reproduction of this work has been made available on figshare^18^ (see Data Records, Table 1). To facilitate usage, the structure and organization of the reported database has also been provided (see Supplementary Table S1).

**Table 1:** List of files encompassing the datasets and the trained model described in this work that are available on figshare^18^.

Filename	Description
coconut_smiles_nostereo_training80.txt	Training and validation dataset of 325,535 unique canonical SMILES without stereochemistry from COCONUT database, January 2022 version (Accessed on 1 August 2022)
coconut_smiles_nostereo_heldout20.txt	Held-out test dataset of 81,384 unique canonical SMILES without stereochemistry from COCONUT database, January 2022 version (Accessed on 1 August 2022)
coconut_rnn_model.pt	Trained RNN model
100million_sampled_smiles.smi	100 million generated natural product-like SMILES sampled from trained RNN model
67M_generated_analysed.json	Json file of 67,064,204 unique canonical generated SMILES with molecular descriptors

As an indication of its cost efficiency, the total computation time for training and sampling was less than 24 hours on an Intel 8268 48-Cores @ 2.9 GHz Nvidia V100 (VRAM = 32 GB and RAM = 192 GB) compute node. A price estimate for similar computing resources on Amazon Web Services (https://calculator.aws/, accessed 23 March 2023) – 24 hours of an dedicated instance (Amazon EC2, c5n.18xlarge instance, 72 vCPUS, 192 GiB memory, Asia-Pacific (Singapore) region, 100 gigabit network performance) would cost USD$155. In comparison, a commercially available 2,576 natural product library is priced two orders of magnitude higher at USD$33,513 (https://www.selleckchem.com/screening/natural-product-library.html, accessed 23 March 2023). Computationally generated natural product databases such as the one reported here are well positioned to push the boundaries of known natural product structures, provide expanded search spaces, and act as a key enabling resource to progress the next generation of *in silico* high throughput screening methods for natural product discovery.

## Methods

### Molecule generation

All software programs were implemented in Python (v3.6.10) with PyTorch (v1.1.0) on an Intel 8268 48-Cores @ 2.9 GHz Nvidia V100 (VRAM = 32 GB and RAM = 192 GB) compute node running on an RHEL 8.3 operating system. The details of all other dependencies can be found in the following environment.yml file (https://github.com/SIBERanalytics/Natural-Product-Generator/blob/master/environment.yml). The generative model was trained with a recurrent neural network (RNN) architecture using long-short-term-memory (LSTM) units (https://github.com/skinnider/low-data-generative-models). To assemble the training and held out datasets, the COCONUT collection of open natural products (https://coconut.naturalproducts.net/, accessed 1 Aug 2022)^11^ was filtered to remove invalid SMILES and take away stereochemistry. This filtered COCONUT dataset was then split into 3 portions, 292,981 (72%) for training, 32,554 (8%) for validation, and 81,384 (20%) as a held-out dataset for testing. The combined training and validation dataset (80% of filtered COCONUT dataset) was augmented by 10 times with their respective non-canonical SMILES using SmilesEnumerator (http://github.com/EBjerrum/SMILES-enumeration) prior to RNN training. This has been shown to improve the validity of the SMILES sampled from the trained model^24^. Determination of the vocabulary of the known natural products was carried out by deconstructing SMILES strings into elemental tokens (e.g. C, N, S, O, c, n, 1, 2..etc). The network consists of 3 layers of RNN with a hidden layer dimension of 512 and no dropout. Training of the network was done with a batch size of 128, a learning rate of 0.001, Adam optimizer, and max epochs set at 1,000. Early stopping patience of 10,000 minibatches was employed. A total of 100,000,000 SMILES strings were sampled from the trained model (with best validation loss of 0.55) after completion of model training.

### RDKit and ChEMBL chemical curation pipeline processing

Data processing was performed using python packages RDKit^36^ (v2020.09.1.0) and chembl_structure_pipeline (v1.0.0) (https://github.com/chembl/ChEMBL_Structure_Pipeline). Generated SMILES strings were converted to canonical SMILES, InChI, and InChIKey molecular representations by sequential application of RDKit functions Chem.MolFromSmiles followed by Chem.MolToSmiles, Chem.inchi.MolToInchi or Chem.inchi.MolToInchiKey respectively. SMILES strings were considered syntactically invalid if no valid molecular representation was returned from either Chem.MolFromSmiles, Chem.MolToSmiles, Chem.inchi.MolToInchi or the Chem.inchi.MolToInchiKey operation. Unique molecular representations, whether canonical SMILES, InChI or InChIKey, were identified by creating a dictionary from the respective molecular representations using the dict.fromkeys(molecular representation) command. Unique generated molecules were then converted to molblock with RDKit function Chem.MolToMolblock before being passed through the ChEMBL structure pipeline to sequentially (1) check for structure quality using checker.check_molblock, (2) standardize structures with chembl_structure_pipeline.standardize_molblock and finally, (3) get parent structures by removing isotopes, salts and solvents with standardizer.get_parent_molblock. Structures returning checker penalty scores of more than 5 were removed. The maximum error score (Max_Error_Score) and the error types (Error_Type) for each remaining entry were recorded. 27 RDkit molecular descriptors (BalabanJ, BertzCT, NumAromaticRings, HallKierAlpha, Kappa1, Chi0, Chi0n, Chi0v, MolLogP, MolMR, MolWt, ExactMolWt, HeavyAtomCount, HeavyAtomMolWt, NHOHCount, NOCount, NumHAcceptors, NumHDonors, NumHeteroatoms, RingCount, FractionCSP3, TPSA, LabuteASA, NumRotatableBonds, NumValenceElectrons, NumSaturatedRings, NumAliphaticRings) from the were calculated and appended for each remaining entry.

#### NPScore and NPClassifier annotations

Natural product-likeness scores (NP_score)^42^ for each generated molecule were calculated using npscorer (https://github.com/rdkit/rdkit/tree/master/Contrib/NP_Score). Natural product pathway (pathway), superclass (superclass), and class (class_type) classifications were assigned using NPClassifier API (https://npclassifier.ucsd.edu/)^43^. Queries without outputs from NPClassifier were assigned the value “none”. Percentage population of generated database receiving value “none” – pathway (11.6%), superclass (40.0%), class (51.1%).

### Kullback-Leibler (KL) Divergence

A measure of the statistical distance between the property probability distributions of known natural product SMILES and generated natural product-like SMILES were calculated with SciPy (v1.7.3) using the function scipy.special.rel_entr(P,Q). This is also described by the following equation:

$$Kullback-Leibler\left(KL\right)\;Divergence{\rm{,}}{D}_{KL}\left(P{\rm{| | }}Q\right)=\sum P\left(x\right)\left(\log \frac{P\left(x\right)}{Q\left(x\right)}\right)$$

Where, P(x) = probability of known natural product SMILES having value x for a given property and Q(x) = probability of generated natural product-like SMILES having value x for a given property.

*NOTE: summation is done across all the possible discrete values of the property (e.g. NPClassifer pathways) where P(x) > 0. In the case where values are in a continuum (i.e. NPScore), ranges of width 0.1 were taken as discrete values*.

### Visualisation of physiochemical and structural space coverage

T-distributed stochastic neighbor embedding (t-SNE) dimensionality reduction was performed on 10 RDkit descriptors (NumAromaticRings, NumAliphaticRings, MolLogP, MolWt, NumHDonors, NumHAcceptors, NumHeteroatoms, TPSA, NumRotatableBonds, and NumValenceElectrons) using scikit-learn (v0.23.2)^48^ function sklearn.manifold.TSNE with the following parameters: n_components = 2, init = “pca”, random_state = 7. Seaborn (v0.11.2) histplot function was used with the following parameters: bins = 50, vmin = 0, vmax = 100,000 to generate structural density maps from the t-SNE data of the generated and known SMILES.

## Data Records

The 67,064,204 natural product-like compound database generated via molecular language processing in this work has been deposited on figshare (Table 1)^18^. The database is organized in a single, two-dimensional array flat model format where elements in each column are the same type of data for a given molecular descriptor and elements in the same row relate to the same molecule. There are a total of 38 columns (i.e. 38 descriptors for each molecule) and 67,064,204 rows (i.e. 67,064,204 molecules in the database). The column numbering, names, data types, and descriptions are listed in Supplementary Table S1.

## Technical Validation

### Testing of generated natural product-like molecules

From the 406,919 known, valid, unique, canonical, natural product SMILES strings in the COCONUT^11^ database with stereochemistry removed, 81,384 (20%) were held-out and the remaining 325,535 (80%) were used to train and validate the recurrent neural network to generate natural product-like SMILES. Of the 81,384 known natural products that were held out as a test set from the training dataset, 30,229 (37% of held-out set) known natural products were reproduced in the generated natural product-like SMILES database, confirming the trained model can generate actual natural product molecules. In addition, the natural product likeness scores (NP Score)^42^ and NPClassifier^43^ pathway distributions of the generated natural product-like molecules have low KL divergence scores of 0.064 and 0.047 nats respectively when referenced against the observed distributions of known natural products from the COCONUT database^11^, indicating that natural product-like molecules have been generated.

## Usage Notes

This generated natural product-like SMILES database covering novel physiochemical and structural space may serve as starting points for high throughput *in silico* discovery of functional natural products. Aside from potential food, agrochemical, and therapeutic applications, there has been increasing consumer demand for natural product alternatives to synthetic ingredients for their perceived health and wellness benefits^49,50^. Such natural alternatives are also amenable to sustainable manufacturing processes via synthetic biology approaches^51,52^, adding to their attractiveness as an answer from chemical manufacturers to environmental regulators^53^ on issues of climate change, pollution, and resource depletion^54^.

## Supplementary information

Supplementary Table S1

## Data Availability

Code used to train the molecular language model as well as the trained model used for natural product-like molecule generation is available from GitHub at https://github.com/SIBERanalytics/Natural-Product-Generator.

## References

[1] Ghirga F (2021). A unique high-diversity natural product collection as a reservoir of new therapeutic leads. Org. Chem. Front..

[2] Zabolotna Y (2021). NP Navigator: A New Look at the Natural Product Chemical Space. Mol. Inf..

[3] Yan Y, Liu Q, Jacobsen SE, Tang Y (2018). The impact and prospect of natural product discovery in agriculture. EMBO Rep..

[4] González-Manzano S, Dueñas M (2021). Applications of Natural Products in Food. Foods.

[5] Lourenço SC, Moldão-Martins M, Alves VD (2019). Antioxidants of Natural Plant Origins: From Sources to Food Industry Applications. Molecules.

[6] Newman DJ, Cragg GM (2016). Natural Products as Sources of New Drugs from 1981 to 2014. J. Nat. Prod..

[7] Stone S, Newman DJ, Colletti SL, Tan DS (2022). Cheminformatic analysis of natural product-based drugs and chemical probes. Nat. Prod. Rep..

[8] Atanasov AG (2021). Natural products in drug discovery: advances and opportunities. Nat. Rev. Drug Discovery.

[9] Shen B (2015). A New Golden Age of Natural Products Drug Discovery. Cell.

[10] Roemer T (2011). Confronting the Challenges of Natural Product-Based Antifungal Discovery. Chem. Biol..

[11] Sorokina M, Merseburger P, Rajan K, Yirik MA, Steinbeck C (2021). COCONUT online: Collection of Open Natural Products database. J. Cheminform..

[12] Koehn FE, Carter GT (2005). The evolving role of natural products in drug discovery. Nat. Rev. Drug Discovery.

[13] Bilodeau C, Jin W, Jaakkola T, Barzilay R, Jensen KF (2022). Generative models for molecular discovery: Recent advances and challenges. WIREs Comput. Mol. Sci..

[14] Yang K (2019). Analyzing Learned Molecular Representations for Property Prediction. J. Chem. Inf. Model..

[15] Martinelli DD (2022). Generative machine learning for de novo drug discovery: A systematic review. Comput. Biol. Med..

[16] Brown N (2020). Artificial intelligence in chemistry and drug design. J. Comput. Aided Mol. Des..

[17] Wilbraham L, Mehr SHM, Cronin L (2021). Digitizing Chemistry Using the Chemical Processing Unit: From Synthesis to Discovery. Acc. Chem. Res..

[18] Tay DWP, Yeo NZX, Adaikkappan K, Lim YH, Ang SJ (2023). figshare.

[19] Harvey AL, Edrada-Ebel R, Quinn RJ (2015). The re-emergence of natural products for drug discovery in the genomics era. Nat. Rev. Drug Discovery.

[20] Vogt M (2022). Using deep neural networks to explore chemical space. Expert Opin. Drug Discovery.

[21] Berenger F, Tsuda K (2021). Molecular generation by Fast Assembly of (Deep)SMILES fragments. J. Cheminform..

[22] Gómez-Bombarelli R (2018). Automatic Chemical Design Using a Data-Driven Continuous Representation of Molecules. ACS Cent. Sci..

[23] Kusner, M. J., Paige, B. & Hernández-Lobato, J. M. in *Proceedings of the 34th International Conference on Machine Learning* **Vol. 70** (eds Precup, D. & Teh, Y. W.) 1945–1954 (PMLR, Proceedings of Machine Learning Research, 2017).

[24] Skinnider MA (2021). A deep generative model enables automated structure elucidation of novel psychoactive substances. Nat. Mach. Intell..

[25] Grisoni F, Moret M, Lingwood R, Schneider G (2020). Bidirectional Molecule Generation with Recurrent Neural Networks. J. Chem. Inf. Model..

[26] Kotsias P-C (2020). Direct steering of de novo molecular generation with descriptor conditional recurrent neural networks. Nat. Mach. Intell..

[27] Prykhodko O (2019). A de novo molecular generation method using latent vector based generative adversarial network. J. Cheminform..

[28] Kadurin A, Nikolenko S, Khrabrov K, Aliper A, Zhavoronkov A (2017). druGAN: An Advanced Generative Adversarial Autoencoder Model for de Novo Generation of New Molecules with Desired Molecular Properties in Silico. Mol. Pharmaceutics.

[29] Lee YJ, Kahng H, Kim SB (2021). Generative Adversarial Networks for De Novo Molecular Design. Mol. Inf..

[30] Sanchez-Lengeling B, Aspuru-Guzik A (2018). Inverse molecular design using machine learning: Generative models for matter engineering. Science.

[31] Weininger D (1988). SMILES, a chemical language and information system. 1. Introduction to methodology and encoding rules. J. Chem. Inf. Comput. Sci..

[32] Moret M, Friedrich L, Grisoni F, Merk D, Schneider G (2020). Generative molecular design in low data regimes. Nat. Mach. Intell..

[33] Brown N, Fiscato M, Segler MHS, Vaucher AC (2019). GuacaMol: Benchmarking Models for de Novo Molecular Design. J. Chem. Inf. Model..

[34] Skinnider MA, Stacey RG, Wishart DS, Foster LJ (2021). Chemical language models enable navigation in sparsely populated chemical space. Nat. Mach. Intell..

[35] Mori K (2011). Bioactive natural products and chirality. Chirality.

[36] RDKit: Open-source cheminformatics; http://www.rdkit.org.

[37] Liu Z, Zubatiuk T, Roitberg A, Isayev O (2022). Auto3D: Automatic Generation of the Low-Energy 3D Structures with ANI Neural Network Potentials. J. Chem. Inf. Model..

[38] Kim Y, Kim WY (2015). Universal Structure Conversion Method for Organic Molecules: From Atomic Connectivity to Three-Dimensional Geometry. Bull. Korean Chem. Soc..

[39] Li Y, Zhou X, Liu Z, Zhang L (2018). Designing natural product-like virtual libraries using deep molecule generative models. J. Chin. Pharm. Sci..

[40] Yu MJ (2011). Natural Product-Like Virtual Libraries: Recursive Atom-Based Enumeration. J. Chem. Inf. Model..

[41] Bento AP (2020). An open source chemical structure curation pipeline using RDKit. J. Cheminform..

[42] Ertl P, Roggo S, Schuffenhauer A (2008). Natural Product-likeness Score and Its Application for Prioritization of Compound Libraries. J. Chem. Inf. Model..

[43] Kim HW (2021). NPClassifier: A Deep Neural Network-Based Structural Classification Tool for Natural Products. J. Nat. Prod..

[44] Brecher J (2006). Graphical representation of stereochemical configuration (IUPAC Recommendations 2006). Pure Appl. Chem..

[45] Bremser W (1978). Hose — a novel substructure code. Anal. Chim. Acta.

[46] Rogers D, Hahn M (2010). Extended-Connectivity Fingerprints. J. Chem. Inf. Model..

[47] Wildman SA, Crippen GM (1999). Prediction of Physicochemical Parameters by Atomic Contributions. J. Chem. Inf. Comput. Sci..

[48] Pedregosa F (2011). Scikit-learn: Machine learning in Python. JMLR.

[49] Asioli D (2017). Making sense of the “clean label” trends: A review of consumer food choice behavior and discussion of industry implications. Food Res. Int..

[50] Maruyama S, Streletskaya NA, Lim J (2021). Clean label: Why this ingredient but not that one?. Food Qual. Prefer..

[51] Scown CD, Keasling JD (2022). Sustainable manufacturing with synthetic biology. Nat. Biotechnol..

[52] Yadav VG, De Mey M, Giaw Lim C, Kumaran Ajikumar P, Stephanopoulos G (2012). The future of metabolic engineering and synthetic biology: Towards a systematic practice. Metab. Eng..

[53] Yi M, Wang Y, Yan M, Fu L, Zhang Y (2020). Government R&D Subsidies, Environmental Regulations, and Their Effect on Green Innovation Efficiency of Manufacturing Industry: Evidence from the Yangtze River Economic Belt of China. Int. J. Environ. Res. Public Health.

[54] Vogel, D. *Trading up: Consumer and environmental regulation in a global economy*. (Harvard University Press, 2009).

